# Oncology Information System: A Qualitative Study of Users’ Requirements

**DOI:** 10.31557/APJCP.2019.20.10.3085

**Published:** 2019

**Authors:** Azadeh Yazdanian, Haleh Ayatollahi, Azin Nahvijou

**Affiliations:** 1 *School of Allied Medical Sciences, Mazandaran University of Medical Sciences, Sari, *; 2 *Health Management and Economics Research Center,*; 3 *Department of Health Information Management, School of Health Management and Information Sciences, Iran University of Medical Sciences,*; 4 *Cancer Research Center of Cancer Institute, Tehran University of Medical Sciences, Tehran, Iran. *

**Keywords:** Clinical information system, neoplasm, oncology information system, cancer informatics

## Abstract

**Background:**

Cancer care is a complex care process and is associated with generating a variety of data during the care process. Therefore, it seems that designing and using information systems is necessary to enhance the accessibility, organization and management of cancer-related data. The aim of this study was to identify users’ requirements of an oncology information system (OIS).

**Methods:**

This was a qualitative study conducted in 2018. In depth semi-structured interviews were performed with clinicians and non-clinicians in five teaching hospitals to identify users’ requirements. Data were analyzed by using framework analysis.

**Results:**

The four themes emerged from data analysis included: a) methods of recording cancer data in the hospitals, b) required cancer data in different departments, c) comprehensive cancer care documentation, and d) required functions of an oncology information system.

**Conclusion:**

According to the results, currently, electronic documentation is less frequently used for cancer patients. Therefore, an extensive effort is needed to identify users’ requirements before designing and implementing an oncology information system. As multidisciplinary teams are involved in cancer care, all potential users and their requirements should be taken into account. Such a system can help to collect and use cancer data effectively.

## Introduction

Cancer is a serious disease characterized by uncontrolled cell division and the invasion of local tissues (Amereh et al., 2016). The global cancer burden increased to 18.1 million new cases and 9.6 million deaths in 2018. Cancers of the lung, female breast, and colorectum are the top three cancer types in terms of incidence, and are among top causes of mortality. Moreover, these three cancer types are responsible for one third of the cancer incidence and mortality burden across the world (World Health Organization, 2018). 

Since early diagnosis and effective treatment can help to increase the survival rate of cancer patients, many different cancer treatment methods have been developed to increase the survival rate (Öhlin, 2016). However, the development of various diagnostic and therapeutic methods for cancer care is subject to data collection and data analysis. Given the increasing volume of cancer data, the use of oncology information systems seems to be inevitable (Han et al., 2005). Moreover, these systems can help to facilitate clinical activities (Heins et al., 2011).

According to the literature, the use of information systems in cancer hospitals, radiotherapy and chemotherapy departments has a number of benefits, such as increasing accessibility of information at the point of need, improving quality of clinical care, reducing medical errors, reducing time of clinical documentation, facilitating outcome reporting, maintaining confidentiality, improving resource allocation, increasing user satisfaction and providing rich data for clinical trials (Mishuris and Linder, 2014; Prokosch et al, 2011). For example, these systems can help to reduce adverse effects of drugs and improve patient safety by providing adequate information about drug dosage during cancer treatment (Bijugn et al, 2014). 

Among different information systems suggested to manage cancer data, oncology information system (OIS) have been suggested to be used in cancer hospitals. An oncology information system supports the provision of integrated care and long-term treatment for cancer patients by collecting data during various stages of cancer care which includes screening, prevention, diagnosis, treatment, palliative care and end-of-life care (Kukreti, 2014). Such a system will also contribute to promote care delivery and support decision-making processes at different stages of cancer care, such as diagnosis, treatment, and follow-up (Kukreti, 2014; Urda et al., 2013). The ultimate goal of oncology information system is to integrate and systematize daily activities in cancer department, to integrate patient data and to utilize statistical tools (Urda et al., 2013). Even after treatment, cancer data are needed for patient follow up. Furthermore, collecting accurate data about cancer patients can help to identify and implement proper interventions against cancer (Gherian et al., 2008).

Although many studies have been conducted to show the application of information systems in the field of oncology, especially radiotherapy (Ando, 2014), studies related to the design of oncology information systems are very limited. For example, Ando designed an oncology information system that supported order entry, managing and presenting a treatment plan, recording treatment outcomes, and storing data. In this system, some standards, such as the Digital Imaging and Communications in Medicine - Radiation Therapy (DICOM-RT), the Integrated Healthcare Enterprise (IHE) and the Health Level 7 (HL7) were used to transfer electronic data between the oncology information system and other hospital systems (Ando, 2014).

In another study, conducted by Evans et al., (2014) in Canada, a geographic oncology information system was implemented in four healthcare organizations. However, the organizational culture and processes were different in these organizations and they encountered a lot of challenges when implementing a standard approach to use the system. The findings of this study showed that paying more attention to work processes, selecting appropriate technologies and defining new workflows should be considered for successful system implementation (Evans et al., 2014). 

In another study, an oncology information system was developed by Urda et al., (2013) in Spain and improved accessibility of patient information. Availability of data analytics tools, integration of the system with clinical workflows, and ability to transfer data to other systems were some of the functions considered in this system. Similarly, Hara and Ikushima (2012) implemented a widespread oncology information system in Japan which was able to share patient data with other departments and supported automatic data entry. 

In Iran, the most common system used for collecting patient data including cancer patients is hospital information systems. Although a national cancer registry programme has been implemented, it does not cover all cases (Zendehdel et al., 2010), and there are a number of problems with incomplete data, getting access to data, the lack of system integration and information sharing between care givers. (Mohammadi et al., 2016) Therefore, it seems that developing an oncology information system can help to improve the accessibility of information and can lead to better use of cancer-related data. As user involvement is a necessary component of designing a successful system, the aim of this study was to identify users’ requirements of an oncology information system.

## Materials and Methods

This was a qualitative research conducted in 2018. Before collecting data, ethical approval was obtained from the Institutional Review Board of Iran University of Medical Sciences. The participants of the study were clinical and non-clinical staff who worked in five teaching hospitals. These teaching hospitals had an oncology department and all potential participants were experienced in caring for cancer patients and recording their data (N=27). Clinical staff included oncologists, radiotherapists, pathologists, chemotherapists and head nurses who worked in the oncology department, and non-clinical participants were health information managers. In depth semi-structured interviews were conducted to collect participants’ perspectives. In order to conduct the interviews, an interview guide was developed based on the literature review (Kukreti et al., 2014; Ando, 2014; Urda et al., 2013; Heins et al., 2011). The interview guide included 11 general questions about the current state of recording cancer-related data, required data for cancer care in different departments and different phases of cancer care, and required functions of an oncology information system. Interviews were recorded and written down in some cases when the interviewee did not permit to record her/his voice. The interviews were fully transcribed and framework analysis method was used to analyze data. This method includes five steps: familiarization, identification of a thematic framework, indexing, charting and mapping and interpretation and is used in applied research with the aim of obtaining information and providing specific recommendations related to the purpose of the study (Srivastava and Bruce, 2009; Ritchie and Liz, 2002). The software used for data analysis was MAXQDA (version 12).

## Results

In this study, 25 out of 27 eligible participants took part in the interviews. The average length of interviews was 40 minutes. The demographic characteristics of the participants are presented in Table 1.

As shown in Table 1, most of the participants (n=17, 68%) were female and the highest frequency (n=11, 44%) was related to the age range of 40-49 years old. Regarding the education level, the highest frequency (n= 12, 48%) was related to those who had a bachelor’s degree. The four themes emerged from the qualitative study are summarized in Table 2. 

*Theme 1. Methods of recording cancer data in the hospitals*

The findings showed that computer-based and paper-based records both were used in the hospitals. The majority of interviewees stated that cancer patients’ data are usually collected and recorded manually by using paper-based records. An oncologist stated that “*a physician records all patient information, such as health status, medical history and clinical history, manually and on the paper.*” (M 3)

In some hospitals, the interviewees noted that patient information should be stored in the hospital information system in addition to being kept as paper records. In this regard, one of the nurses said: “*some patient information, including laboratory data and imaging should also be recorded in the hospital information system in addition to being filed in a paper file.*” (M 17)

In some hospitals, patient information was first recorded on the paper and then, it was scanned. However, the pathology tests results were all recorded in the hospital information system.

*Theme 2. Required cancer data in different departments*

Since cancer patients are treated in different departments, the required data in each department might be vary from other departments. Three main departments involved in cancer care were oncology department, radiotherapy department and chemotherapy department. 

*Oncology department*

The required data for the oncology department were summarized in five sub-categories, including individual data, initial assessment data, diagnostic and treatment data, disease progress report, and discharge data. The majority of interviewees stated that the required individual data are age, sex, patient’s address, patient’s social and economic status, height, weight, and body mass index. One of the oncologists said: “*knowing the socioeconomic status of a cancer patient is very important because when we know where the chemotherapy takes place, we identify the medications we can give them, and ultimately the patient will be able to pay the cost.*” (M 5) According to the results, initial assessment data included physical and clinical examinations, patient and family medical history, history of drug use, and food and drug allergy. One of the nurses explained that “*in the initial assessment, there are many things that can be done, including possible history of surgery, history of miscellaneous medications, family history, nursing diagnosis, and many more items, such as having a history of pain and ulcer, drug interactions, food and drug allergies.*” (M 18)

Most of the interviewees stated that diagnostic and treatment data included the laboratory tests, ultrasounds, CT scans, MRI results as well as the pathology report. These results confirm the final diagnosis, tumor stage and metastatic sites which are necessary data for a cancer patient. Regarding treatment data, the details of chemotherapy, radiotherapy, surgical procedures, hormone therapy, and immunotherapy were found to be important. A disease progress report including the details of daily visits, physician’s orders, nursing and counseling reports, patient discharge data and follow-up plans were also found to be important from the interviewees’ point of view.

*Radiotherapy department*

The interviewees stated that a variety of data were collected in the radiotherapy department. These data was summarized in three subcategories of patient consent, medical history, and radiotherapy data. Most of the interviewees explained that the patient must be mentally prepared before radiotherapy and possible barriers and obstacles must be removed. For example, one of the nurses said: “*patient satisfaction is very important for radiation therapy. If the patient is unwilling, the written consent will be obtained from his/her family.*” (M 17)

The majority of the interviewees stated that various information in a medical history need to be reviewed before radiotherapy. For example, final diagnosis, interventions, laboratory tests, ultrasound, MRI and CT scan results, biopsy and surgery, pathology result including the details of cellulogy, grade and stage of the tumor, and recommended treatment processes are important for a radiotherapist. Moreover, it is important to know whether the patient is supposed to undergo radiotherapy or radiotherapy and chemotherapy will be used together. As sometimes radiotherapy is performed prior to surgery to reduce the mass and sometimes it is used after surgery to treat the patient, it is also important to know what type of radiotherapy is required. Other necessary data included radiation dosage, treatment location, treatment division, and type of the radiation.

**Table 1:** Demographic Characteristics of the Participants

Variable	Frequency (%)
Sex	
Male	8 (32%)
Female	17 (68%)
Age	
30-39	5 (20%)
40-49	11 (44%)
50-59	9 (36%)
Education	
B.Sc.	12 (48%)
M.Sc.	3 (12%)
Medical Specialist	10 (40%)
Field of study	
Oncology	4 (16%)
Radiation therapy	3 (12%)
Pathology	3 (12%)
Nursing	10 (40%)
Health Information Management	5 (20%)
Work experience	
1-5	3 (12%)
6-10	4 (16%)
11-15	8 (32%)
16 years and more	10 (40%)

**Table 2:** Themes, Categories and Subcategories

Theme	Category	Sub-category
Methods of recording cancer data in the hospitals	Computer-based records Paper-based records	
Required cancer data in different departments	Oncology department	Individual data Initial assessment data Diagnosis and treatment data Disease progress report Discharge data
	Radiotherapy department	Patient consent Medical history Radiotherapy data
	Chemotherapy department	Medical history Laboratory tests results Chemotherapy data
Comprehensive cancer care documentation	Cancer screening	Various data for different types of cancer
	Cancer prevention	Various data for early detection and prevention of cancer Various data for cancer recurrence prevention
	Cancer diagnosis	Patient history Laboratory tests results
	Cancer care	Medical care data Nursing care data
	Mental health and pain relief	Psychiatric care data Supportive care data
	End of life care	Spiritual care data Supportive care data
Required features of an oncology information system	Possibility of searching data Security and confidentiality protocols Data analytic tools Collecting needed data	

*Chemotherapy department*

The necessary data in the chemotherapy department were divided into three sub-categories of medical history, laboratory tests results, and chemotherapy data. Most of the interviewees stated that patient’s medical history, other illnesses, and medications are very important in chemotherapy. For example, one of the radiotherapists said: “*since chemotherapy has a side effect that may affect other diseases, knowing underlying illnesses is very important.*” (M 7)

Other interviewees noted that before chemotherapy, some laboratory tests, such as complete blood cell and biochemistry need to be performed and the blood samples must be taken either on the same day or the day before chemotherapy.

Regarding the chemotherapy data, the name of the drug, dosage, the method of administrating the drug, the date of treatment and referral, the complications that may occur, the number of injection courses and the interval between injections were found to be important data in chemotherapy. Moreover, an oncologist said: “*each type of cancer has a particular chemotherapy diet that is determined by the patient’s age and clinical condition.*” (M 2)

*Theme 3. Comprehensive cancer care documentation*

In order to support comprehensive cancer care documentation, data related to different phases of cancer care need to be collected. These phases are cancer screening, prevention, diagnosis, care, mental health and pain relief and end-of-life care which are discussed below.

*Cancer screening*

The interviewees stated that different types of data were collected for screening a variety of cancers. Most interviewees stated that screening programs vary from country to country, and only common cancers in each country are screened. The interviewees stated that the required data for screening various types of cancers included demographic data such as age, gender, weight, height, occupational history, family history, smoking status, medication history, the history of pregnancy and radiation exposure at that time, chemical exposure, nutritional status, and the screening tests results. Moreover, the interviewees said that screening tests were different for different types of cancer. For example, colonoscopy is used for colorectal cancer and Pap smear test is considered for cervical cancer.

*Cancer prevention*

According to the results, cancer prevention could be categorized into early detection and prevention of cancer and cancer recurrence prevention. To prevent the disease, data similar to the screening data could be useful. However, to prevent cancer recurrence, periodic laboratory tests, chemotherapy history, radiotherapy history or the history of being exposed to other types of radiations, and medications history were found to be essential.

*Cancer diagnosis*

The interviewees stated that medical history and laboratory tests results are necessary for cancer diagnosis. Medical history included symptoms, such as sever fever, sever weight loss, anorexia, long-term headaches, bone pain, changes in bowel habits, frequent bleeding, chronic ulcers, pallor, coughing and cramps, spontaneous bruising, colds that do not recover and any other unpleasant psychological and physical symptoms. Moreover, risk factors such as smoking and drinking alcohol as well as occupational risk factors need to be considered. The main clinical tests included ultrasound, CT scan, MRI, and pathology. A radiotherapist said: “*cancer diagnosis varies case by case, but the most important tool is the patient’s pathology report.*” (M 6)

*Cancer care*

Cancer care data were summarized in medical and nursing care data. The interviewees noted that the necessary data for medical care were related to surgery, radiotherapy, chemotherapy, hormone therapy and immunotherapy data. A number of interviewees also stated that the needed data for nursing care are related to controlling fever, vital signs, disease symptoms, and the level of consciousness. One of the nurses said: “*nursing care includes simple care, such as controlling vital signs, skin changes, and in some cases, limited mental healthcare.*” (M 23)

*Mental health and pain relief*

Most interviewees believed that, despite the great importance of mental health and pain relief, unfortunately, this type of care is not provided in the cancer centers of the country. However, these data are important and can be summarized in two sub-categories of psychiatric care data and supportive care data. Interviewees believed that cancer patients need mental and supportive care from the very first days of diagnosis. Psychiatric data can be generated by both psychiatrists and psychologists. Moreover, psychiatric care and education are needed at the time of discharge to help the patient to return to the society and her/his daily life. 

Regarding the supportive care, the interviewees believed that cancer patients needed pain relief and nutritional supplements from the beginning of treatment. An interviewee noted that since pain is a risk factor for suicide, it should be precisely monitored. In supportive care it is important to know: is the patient suffering from any form of pain? How much pain did the patient have? Has the pain been monitored? What has been done for the patient?

*End-of-life care*

Many interviewees believed that cancer patients needed special care to improve their life quality at the end of their life, and related data can be summarized in two categories of providing spiritual care to the patient and her/his family and providing supportive care. Most interviewees stated that spiritual care is important for cancer patients in addition to the physical and mental care and it is necessary for both patient and her/his family. In spiritual care, the following questions need to be answered: Does the patient have any problem or spiritual challenge? Has anything been done for her or him? Is the patient’s family experiencing problems or spiritual problems? Has anything been done for them? Moreover, most interviewees believed that patients may need more pain relief medications and their nutritional status needs to be monitored at this stage. Therefore, the related data should be collected and be available for any clinical decision making.

*Theme 4. Required functions of an oncology information system*

Most interviewees believed that functions, such as, the possibility of searching data, security protocols and the availability of data analytic tools were the most common functions that should be considered in an oncology information system. Moreover, they noted that the system should be able to collect all necessary data, including chemotherapy, radiotherapy, surgical and medical data, etc. Given the huge cost of treating cancer patients, the interviewees believed that collecting data for screening and preventing cancers should be taken into account as a priority. In this regard, a radiotherapist said: “*variables that are commonly used for cancer research are not usually recorded. To do this, variables related to the research, including a cancer stage, the degree of malignancy, the patient’s survival rate, and the metastasis sites must be clearly recorded. *“(M 6)

## Discussion

Oncology Information System (OIS) are used to manage cancer data in hospitals and facilitate monitoring and documenting various aspects of cancer care. These systems can help defining clinical workflows, clinical protocols, and structured treatment plans as well as improving patient care and reducing medical errors (Ando, 2014; Evans et al., 2014). However, the results of the current study showed that cancer care data were usually documented manually by using paper-based records and only clinical tests results were electronically available via hospital information systems. In similar studies, the importance of electronic recording of cancer data has been emphasized. For example, the results of Scicotte et al.,’s study (2016) showed that the use of electronic medical records in outpatient cancer centers could help to improve the flow of information and reduced the average waiting time. In another study, the interest of clinicians in using oncology electronic medical records has been highlighted (Ries et al., 2012) or an oncology information system has been developed to record cancer data electronically (Yu et al, 2010). It seems that collecting cancer care data electronically has a number of benefits for patients, clinicians and policy makers and can be regarded as an important tool for improving quality of care, making informed decisions, saving costs and reducing time for patient care. 

According to the findings, and given the importance and details of cancer care data, an oncology information system should be able to cover all needed data from the earliest stages of screening to the latest stages of the disease. The results are in line with the findings of other similar studies which showed that various data, such as disease history, tests results, final diagnosis and cancer stage, long-term adverse effects and outcomes should be recorded for cancer patients (Yu et al., 2010). In a study conducted by Ries et al. (2012), the needed data were extracted from different clinical information systems and were integrated to report the patient’s health status.

In Levy et al.,’s study (2011), a form was developed in the electronic health records system for collecting data related to the chemotherapy protocols, pre-treatment symptoms assessment and patient education. The main data included the name and dosage of the drug, the rate and the method of injection, as well as the expiry date of the drug. In another study, an advanced medical information system database (AMIDAS) was developed for archiving radiotherapy information. In this system, the main collected data were patient demographic information, tumor data, radiotherapy treatment plan, follow-up data (tumor complications, disease progression, mortality, etc.), and laboratory tests results (Mukai et al., 2015). Similarly, Ontario Cancer Care Center created an out-patient electronic medical records system for cancer patients. In this system, all data elements related to cancer screening, prevention, diagnosis, treatment, psychiatric care and pain relief and end-of-life care were recorded (Kukreti, 2014). There are also many other studies in which the importance of using electronic information systems for recording radiotherapy (Mukai et al., 2015; Estathiou et al., 2013; Yang et al., 2012) and chemotherapy data have been highlighted (Levy et al., 2011; Pirnejad et al., 2011). As an oncology information system can support different aspects of cancer care, it seems that developing a complete and comprehensive system can be more efficient for the system end-users. 

According to the results, the main required system functions were the possibility of searching data, security and confidentiality protocols, the availability of data analytic tools and collecting data about different types of cancer. In other studies, the importance of technical requirements before implementing oncology information systems has been highlighted (Ando, 2014). Urda noted that applying DICOM-RT standard in radiotherapy, IHE data integration standard for describing workflows and HL7 standard for the transmission of electronic data between oncology information system and other hospital information systems are necessary (Urda et al., 2013). In another study, maintaining security and confidentiality of data in an oncology information system has been discussed in details (Estathiou et al., 2013). Overall, the results of the research showed that an oncology information system is complex and various data and system functions have to be considered to generate a complete cancer care record. 

*Research limitations*

Although a number of data elements and system functions required to design an oncology information system were identified in this study, the results need to be validated in a bigger sample size by using quantitative methods. Moreover, the results of the current study focused on many details which should be considered at the time of system design. While this level of details can be useful for designing the system, the actual system has to be designed, implemented and evaluated in a real environment to explore whether users’ requirements have been met or not. 

In conclusion, the present study was conducted to identify users’ requirements of an oncology information system. The results showed that cancer care has different stages and patients may receive a verity of healthcare services. Therefore, an oncology information system should be designed to cover all needed data and functions. Such a system can help to improve the quality of care, documentation, research and allocating different resources. However, it is important to see whether these data elements and system functions can meet users’ requirements or not. More research is required to design and evaluate different types of oncology information system (OIS). 
